# The incidence of interstitial lung disease 1995–2005: a Danish nationwide population-based study

**DOI:** 10.1186/1471-2466-8-24

**Published:** 2008-11-04

**Authors:** Jette B Kornum, Steffen Christensen, Miriam Grijota, Lars Pedersen, Pia Wogelius, Annette Beiderbeck, Henrik Toft Sørensen

**Affiliations:** 1 Department of Clinical Epidemiology, Aarhus University Hospital, Aarhus, Aalborg, Denmark; 2 Worldwide Epidemiology, GlaxoSmithKline, Greenford, UK

## Abstract

**Background:**

Current data on incidence of interstitial lung diseases (ILDs) are sparse and concerns about an increasing trend have been raised. We examined incidence rates (IRs) of ILDs and changes in IRs between 1995 and 2005.

**Methods:**

All persons with a first-time hospital discharge or outpatient diagnosis of ILD were identified through the Danish National Registry of Patients, which covers all Danish hospitals. Crude and age-standardised IRs were computed for ILD overall, as well as stratified by ILD subcategories.

**Results:**

A total of 21,765 patients with ILD were identified. Between 1995 and 1998 the overall standardised IR of ILD decreased from 27.14 (95% CI 25.82–28.46) per 100,000 person-years to 19.36 (95% CI 18.26–20.46) per 100,000 person-years. After 1998 the IR increased considerably, peaking at 34.34 (95% CI 32.84–35.85) per 100,000 person-years in 2002. Subsequently there was a slight decrease. The highest IR was observed in the non-specific category "Respiratory disorders in diseases classified elsewhere". By ILD subcategory, the greatest average increase during the study period was observed in "Respiratory disorders in diseases classified elsewhere".

**Conclusion:**

The incidence rate of ILD in Denmark increased during the study period, most pronounced for ILDs associated with systemic diseases.

## Background

Interstitial lung diseases (ILDs) are a heterogeneous group of more than 200 different serious disease entities with common functional characteristics such as restrictive physiology and impaired gas exchange, and with variable degrees of pulmonary inflammation and fibrosis [1,2]. Approximately two-thirds of ILD cases have no reported aetiology [3]. The remaining one-third is associated with or defined by various environmental or occupational factors including cigarette smoking, aspiration, certain drugs, radiation therapy, cancer, and systemic diseases with lung involvement [2-4].

Data on the incidence of ILDs are sparse [2,4]. The few previous studies have reported an eightfold variation in ILD incidence, from 3.62 per 100,000 person-years in southern Spain [5] to 31.5 per 100,000 person-years in males and 26.1 per 100,000 person-years in females in New Mexico, USA [6]. The inconsistency of estimated incidence rates internationally may stem from differences in sampling procedures and diagnostic criteria and measurement bias arising from variation in coding practices and case ascertainment. Previous European studies of ILD occurrence, based on questionnaires sent to chest physicians, may underestimate the true incidence [5,7-10]. The only truly population-based study, conducted over 15 years ago, encompassed a relatively small US population of 480,577 and was restricted to a 2-year study period [6].

While some reports suggest that the prevalence and incidence of ILDs have increased during recent decades [3], no population-based longitudinal studies have documented recent temporal trends in ILD subgroups.

Updated incidence data are essential for predicting health care demand, for evaluating continuing needs for prevention programs such as asbestosis abatement, and to foster our understanding of possible changes in presumed risk factors over time.

We conducted this population-based study to examine the current incidence rate of ILDs and temporal changes in incidence rate between 1995 and 2005.

## Methods

### Setting and study population

The entire Danish population of 5.4 million people was setting for this population-based study [11]. The Danish National Health Service provides tax-supported health care for all residents, including free access to primary care and hospitals. Care of all patients with complicated respiratory diseases, including ILDs, is provided by specialised centres within public hospitals operating under the auspices of the Danish National Health Service. Since 1968, the Danish Civil Registration System has kept electronic records, updated daily, on date of birth, gender, change of address, date of emigration, and changes in vital status for all Danish residents. Use of civil registration numbers, assigned to every Danish resident, allows accurate linkage among Danish registries [11].

### Identification of patients with interstitial lung disease

The Danish National Registry of Patients (NRP) contains information on all discharges from non-psychiatric hospitals since 1977. Information on outpatient and emergency room visits was added in 1995. The NRP includes civil registration number, dates of hospital admission and discharge, and up to 20 discharge diagnoses, classified by physicians according to the International Classification of Diseases, 8^th ^revision (ICD-8) until the end of 1993 and according to the 10^th ^revision (ICD-10) thereafter. We included all patients with a first-time hospital discharge or hospital outpatient visit diagnosis of ILD between 1 January, 1995 and 31 December, 2005. We included ILD diagnoses registered as either primary or secondary diagnosis in the hospital discharge list. The following ICD-10 codes were used: J60.X–J70.X (except J66, J68.2, J70.8, and J70.9), J82.X, J84.X, J99.X, D76.0, D86.0 and D86.2. (See additional file 1 for further information on ICD codes). Since we were interested in the incidence of ILD, we excluded patients diagnosed with ILD in 1994.

### Statistical analysis

We computed annual crude and age-standardised incidence rates (IRs) for ILD overall and stratified by gender. IRs were defined as the number of patients with a first-time diagnosis of ILD in a given year divided by the number of citizens alive in Denmark in the middle of that year (obtained from Statistics Denmark). Age-standardised IRs were computed based on the 2000 world population (age groups: 0–14 years, 15–39 years, 40–64 years, 65–79 years, and 80+ years).

We divided the 10-year study into two periods (1 January, 1995 – 31 December, 2000 and 1 January, 2001 – 31 December, 2005) and defined IRs as the number of patients with a first time diagnosis of ILD in each time period divided by the total number of citizens alive in Denmark in the middle of each calendar year band. This approach was used overall and within subcategories of ICD-10 codes, as well as for gender- and age-specific strata. We then computed age-standardised rates for overall ILDs, gender, and subcategories of ILDs, standardised to the 2000 world population.

Statistical analyses were performed with SAS software (version 9.1.3; SAS Institute, Cary, NC). The study was approved by the Danish Data Protection Agency (Record no. 2005-41-5511). No ethics approval was required because no primary data collection was done.

## Results

### Overall incidence

Between 1995 and 2005, 21,765 patients were registered with a first-time discharge or outpatient diagnosis of ILD. The median age of the 12,639 (58%) men was 63 years (interquartile range 44–75 years) and that of the 9,126 (42%) women was 64 years (interquartile range 45–76 years).

**Figure 1:**
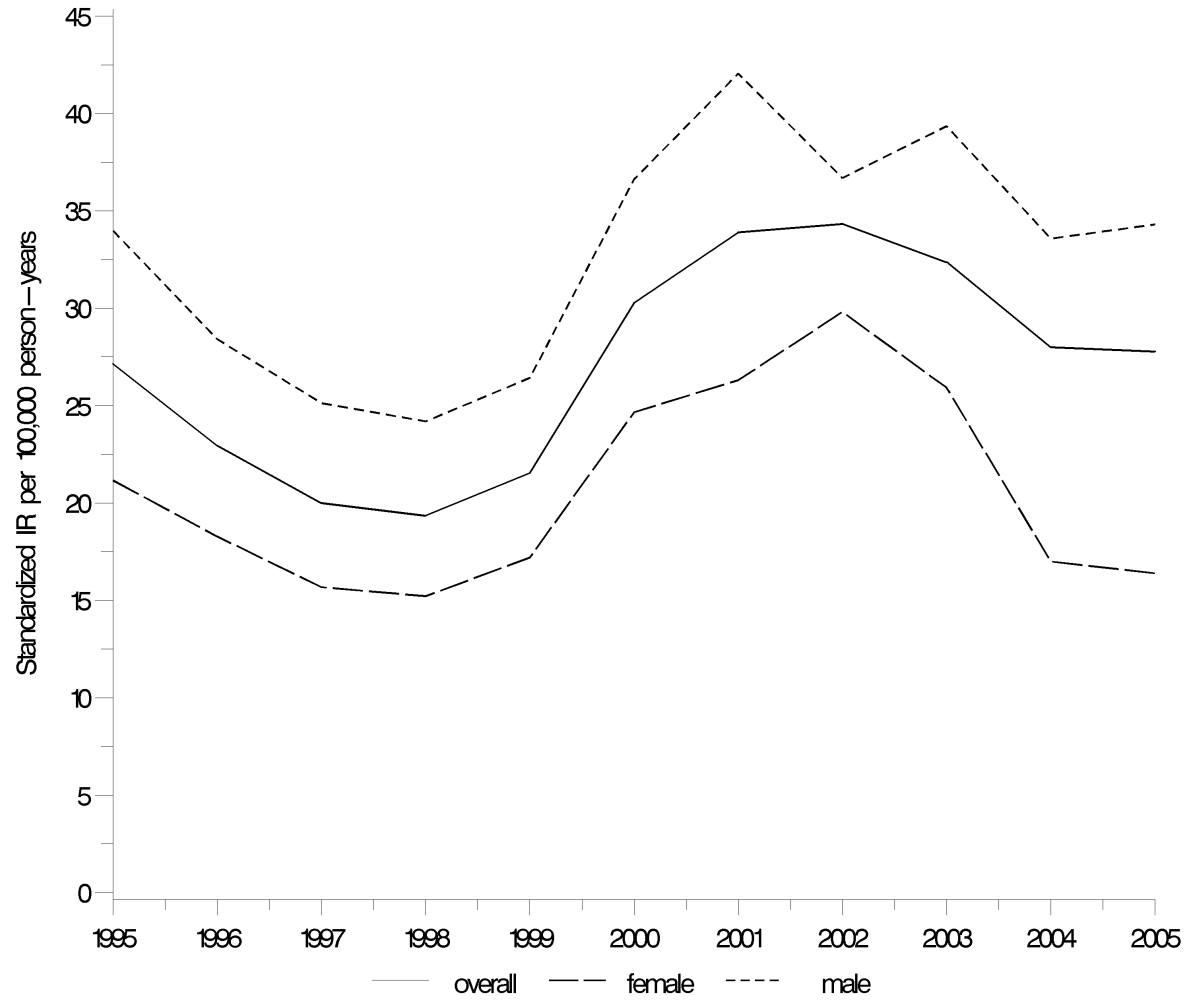
**Standardised incidence rates of ILD overall and by gender. **Denmark 1995–2005. Age-standardised to the 2000 world population.

Figure 1 shows the annual standardised IRs of ILD. Between 1995 and 1998 the overall standardised IR of ILD decreased from 27.14 (95% CI 25.82–28.46) per 100,000 person-years to 19.36 (95% CI 18.26–20.46) per 100,000 person-years. After 1998 the IR increased considerably, peaking at 34.34 (95% CI 32.84–35.85) per 100,000 person-years in 2002. Subsequently there was a slight decrease.

The age-standardised IR increased from 23.54 (95% CI 23.04–24.04) per 100,000 person-years in 1995–2000 to 31.28 (95% CI 30.64–31.92) per 100,000 person-years in 2001–2005 (Table 1). The IR of ILDs increased with increasing age, from 18.90 (95% CI 18.00–19.80) per 100,000 person-years in those aged 15–39 years to 165.41 (95% CI 157.73–173.08) per 100,000 person-years in those aged ≥80 years. Age-standardised incidence rates of ILDs were approximately 50% higher in males than in females throughout the study period.

**Table 1:** The incidence (per 100,000 person-years) of ILD in Denmark overall and within age- and gender-specific strata

**1995–2000**	**Cases** **(N)**	**Incidence rates** **Crude** (95% CI)*	**Incidence rates** **Age-standardised** (95% CI)*
Overall	10,318	32.57 (31.94–33.20)	23.54 (23.04–24.04)
			
Age			
0–14	609	10.76 (9.91–11.62)	
15–39	1,449	13.01 (12.34–13.68)	
40–64	3,312	32.72 (31.61–33.84)	
65–79	3,584	101.81 (98.48–105.15)	
80+	1,364	110.06 (104.21–115.90)	
Gender			
Male	6,063	38.75 (37.78–39.73)	29.12 (28.33–29.90)
Female	4,255	26.54 (25.74–27.33)	18.69 (18.05–19.33)

**2001–2005**			
			
Overall	11,447	42.66 (41.88–43.44)	31.28 (30.64–31.92)
			
Age			
0–14	815	16.23 (15.12–17.35)	
15–39	1,693	18.90 (18.00–19.80)	
40–64	3,737	42.08 (40.74–43.43)	
65–79	3,418	118.02 (114.07–121.98)	
80+	1,784	165.41 (157.73–173.08)	
Gender			
Male	6,576	49.56 (48.36–50.76)	37.79 (36.81–38.78)
Female	4,871	35.90 (34.90–36.91)	25.43 (24.61–26.25)

*CI: confidence interval

### Incidence of specific ILD diagnoses

In the 2001–2005 period, ILD subcategories with the highest IRs were "Respiratory disorders in diseases classified elsewhere" (standardised IR 7.73; 95% CI 7.39–8.07), "Other interstitial pulmonary diseases except idiopathic pulmonary fibrosis" (standardised IR 6.26; 95% CI 6.00–6.52), "Pneumonitis due to solids and liquids" (standardised IR 5.20; 95% CI 4.92–5.47) and "Sarcoidosis with lung involvement" (standardised IR 4.11; 95% CI 3.87–4.34) (Table 2). The highest average increase in incidence between 1995–2000 and 2001–2005 was in the subcategory J99 "Respiratory disorders in diseases classified elsewhere" (Table 2 and 3). The IR for each subcategory of J99 ("J99.0 Rheumatoid lung disease", "J99.1 Respiratory disorders in other diffuse connective tissue disorders" and "J99.8 Respiratory disorders in other diseases classified elsewhere") is also shown in table 2 and 3. Of the 3,152 patients with a J99 diagnosis, 862 were coded with a subcategory of J99. A decrease in incidence between the two periods was found only in the following subcategories: "pneumoconiosis due to asbestos and other mineral fibres, dust containing silica, and other inorganic dust", "unspecified pneumoconiosis", "pulmonary eosinophilia", "idiopathic pulmonary fibrosis".

**Table 2:** Crude and age-standardized incidence (per 100,000 persons-years) within subcategories of ILD in Denmark 2001–2005

**Subcategories and diseases**	**ICD code**	**Cases (N)**	**Incidence rates** **Crude** (95% CI)*	**Incidence rates** **Age-standardised** (95% CI)*
Coalworkers' pneumoconiosis	J60	17	0.06 (0.03–0.09)	0.05 (0.03–0.08)
Pneumoconiosis due to asbestos and other mineral fibres	J61	332	1.24 (1.10–1.37)	0.70 (0.62–0.78)
Pneumoconiosis due to dust containing silica	J62	53	0.20 (0.14–0.25)	0.14 (0.10–0.18)
Pneumoconiosis due to other inorganic dusts	J63	29	0.11 (0.07–0.15)	0.07 (0.04–0.10)
Unspecified pneumoconiosis	J64	173	0.64 (0.55–0.74)	0.36 (0.30–0.42)
Pneumoconiosis associated with tuberculosis	J65	18	0.07 (0.04–0.10)	0.08 (0.04–0.12)
Hypersensitivity pneumonitis due to organic dust	J67	213	0.79 (0.69–0.90)	0.70 (0.60–0.80)
Respiratory conditions due to inhalation of chemicals, gases, fumes and vapours	J68 (except J68.2)	519	1.93 (1.77–2.10)	1.80 (1.64–1.97)
Pneumonitis due to solids and liquids	J69	1,915	7.14 (6.82–7.46)	5.20 (4.92–5.47)
Respiratory conditions due to other external agents	J70 (except J70.8, J70.9)	191	0.71 (0.61–0.81)	0.43 (0.37–0.50)
Pulmonary eosinophilia, not elsewhere classified	J82	161	0.60 (0.51–0.69)	0.39 (0.32–0.45)
Idiopathic pulmonary fibrosis	J84.1	1,417	5.28 (5.01–5.56)	2.91 (2.75–3.08)
Other interstitial pulmonary diseases except idiopathic pulmonary fibrosis	J84 (except J84.1)	2,619	9.76 (9.39–10.13)	6.26 (6.00–6.52)
Respiratory disorders in diseases classified elsewhere	J99	2,461	9.17 (8.81–9.53)	7.73 (7.39–8.07)
Rheumatoid lung disease	J99.0	21	0.08 (0.04–0.11)	0.06 (0.03–0.09)
Respiratory disorders in other diffuse connective tissue disorders	J99.1	46	0.17 (0.12–0.22)	0.12 (0.08–0.16)
Respiratory disorders in other diseases classified elsewhere	J99.8	705	2.63 (2.43–2.82)	2.27 (2.09–2.46)
Langerhans'cell histiocytosis	D76.0	72	0.27 (0.21–0.33)	0.35 (0.27–0.44)
Sarcoidosis with lung involvement	D86.0 orD86.2	1,257	4.68 (4.43–4.94)	4.11 (3.87–4.34)

*CI: confidence interval

**Table 3:** Crude and age-standardized incidence (per 100,000 persons-years) within subcategories of ILD in Denmark 1995–2000

**Subcategories** **and diseases**	**ICD code**	**Cases** **(N)**	**Incidence ** **rates Crude** (95% CI)*	**Incidence rates** **Age-standardised** (95% CI)*
Coalworkers' pneumoconiosis	J60	7	0.02 (0.01–0.04)	0.02 (0.00–0.03)
Pneumoconiosis due to asbestos and other mineral fibres	J61	540	1.70 (1.56–1.85)	1.01 (0.92–1.09)
Pneumoconiosis due to dust containing silica	J62	116	0.37 (0.30–0.43)	0.24 (0.19–0.29)
Pneumoconiosis due to other inorganic dusts	J63	45	0.14 (0.10–0.18)	0.10 (0.07–0.13)
Unspecified pneumoconiosis	J64	229	0.72 (0.63–0.82)	0.41 (0.35–0.47)
Pneumoconiosis associated with tuberculosis	J65	13	0.04 (0.02–0.06)	0.05 (0.02–0.08)
Hypersensitivity pneumonitis due to organic dust	J67	255	0.80 (0.71–0.90)	0.68 (0.59–0.77)
Respiratory conditions due to inhalation of chemicals, gases, fumes and vapours	J68 (except J68.2)	549	1.73 (1.59–1.88)	1.62 (1.48–1.77)
Pneumonitis due to solids and liquids	J69	1,449	4.57 (4.34–4.81)	3.86 (3.63–4.10)
Respiratory conditions due to other external agents	J70 (except J70.8, J70.9)	174	0.55 (0.47–0.63)	0.38 (0.32–0.45)
Pulmonary eosinophilia, not elsewhere classified	J82	299	0.94 (0.84–1.05)	0.63 (0.56–0.71)
Idiopathic pulmonary fibrosis	J84.1	2,303	7.27 (6.97–7.57)	4.17 (3.99–4.36)
Other interstitial pulmonary diseases except idiopathic pulmonary fibrosis	J84 (except J84.1)	2,374	7.49 (7.19–7.80)	4.89 (4.68–5.10)
Respiratory disorders in diseases classified elsewhere	J99	691	2.18 (2.02–2.34)	1.83 (1.68–1.99)
Rheumatoid lung disease	J99.0	4	0.01 (0.00–0.02)	0.01 (0.00–0.02)
Respiratory disorders in other diffuse connective tissue disorders	J99.1	20	0.06 (0.04–0.09)	0.05 (0.03–0.08)
Respiratory disorders in other diseases classified elsewhere	J99.8	66	0.21 (0.16–0.26)	0.19 (0.14–0.24)
Langerhans'cell histiocytosis	D76.0	85	0.27 (0.21–0.33)	0.37 (0.29–0.45)
Sarcoidosis with lung involvement	D86.0 or D86.2	1,189	3.75 (3.54–3.97)	3.27 (3.08–3.46)

*CI: confidence interval

## Discussion

In this large population-based study conducted within a well-defined Northern European population, the overall IR of ILDs was around 31 per 100,000 person-years in 2001–2005 and increased with 33% between 1995–2000 and 2001–2005. The increased incidence was observed in all age groups and in both genders. The increase was most noticeable in ILDs associated with other systemic diseases.

The study's longitudinal population-based design, based on data from the free tax-supported Danish health care system, enabled us to identify all hospital discharge and outpatient diagnoses of ILDs during a 10-year period, and limited the risk of referral and diagnostic bias. The validity of our findings depends ultimately on the accuracy of ILD coding, including levels of diagnostic work-up, and completeness of reporting in the Danish National Registry of Patients. A previous examination of the validity of diagnoses of interstitial lung diseases caused by external agents in the Danish National Registry of Patients yielded high positive predictive values of 92% (95% CI: 73%–98%) for drug induced ILDs and 87% (95% CI: 59%–98%) for radiation induced ILDs, compared with other discharge diagnoses [12,13]. The ICD-10 codes used to identify cases in this study do not correspond completely to the new classification recently developed for ILDs [14]. We thus were not able to apply the new classification to data in the Danish National Registry of Patients.

We may have missed patients with ILD seen only by primary care physicians, causing an underestimation of the true incidence. However, to explain the increasing incidence of ILDs, the number of ILD patients treated by primary care physicians should have changed substantially during the 10 year study period. This is an unlikely scenario, particularly since the Danish National Board of Health recommends that diagnosis and treatment of patients with ILDs occur only at highly specialised national centres [15]. While younger patients with ILD may be more likely to be referred to a hospital for treatment, controlling for age in the analysis limited the potential effect of this bias on our results.

We excluded patients registered with a diagnosis of ILD in 1994, to avoid including prevalent ILD cases. Still, we cannot entirely exclude the possibility that a combination of prevalent and incident cases might have been captured in the beginning of the study period. This potentially could lead to overestimation of ILD incidence in the beginning of the study period and to underestimation of the increase in incidence during the entire period and thus attenuate the time trend changes.

The IR found in our study is almost ninefold higher than that reported for previous European studies [5], perhaps because the earlier studies may have been limited by incomplete reporting restricted to chest physicians' diagnoses [5,8]. Contrary, our results accord with those from the 1994 population-based two-year cross sectional study conducted by Coultas *et al*. in New Mexico [6] despite the use of different case ascertainment methods and patients' exposure to different environmental factors. In the New Mexico study, patients with ILDs were identified from a number of sources: hospital diagnoses, physician referrals, histopathology reports, and death certificates.

Our study is the first to report recent 10-year time trends for ILD subgroups in a defined population. Our findings of a slightly decreased incidence rate of idiopathic pulmonary fibrosis is in contrast to two recent US studies reporting an increased incidence and mortality from idiopathic pulmonary fibrosis [16,17]. This discrepancy could in part be due to geographic variations but also to differences in data collection methods. Based on death certificates diagnoses, classified according to ICD-9 until the end of 1998 and according to ICD-10 thereafter, Olson et al. found increasing mortality rates from pulmonary fibrosis between 1992 and 2003 [16]. They were, however not able to determine, whether this increase at least in part could be explained by differences in coding during the study period. Using a national health care claims database Raghu et al. found that idiopathic pulmonary fibrosis is more common than previous reported but no time trends were presented [17]. In our study, although using the same coding system throughout the study period, we can not entirely exclude the possibility that there may have been changes in coding of diagnoses with a shift from idiopathic pulmonary fibrosis (J84.1) to more disease specific codes."

The rising overall incidence of ILDs could reflect a causal rise, but the use of new and more sensitive diagnostic methods, such as high-resolution CT-scans may have contributed to the rise [3]. In Denmark the number of CT-scans increased during the study period, which may have increased the likelihood of diagnosing mild ILD cases and thus could explain some of the observed rising incidence rate of ILD. Still, the increased use of CT-scans would most likely primarily have influenced diseases classification. However, the large group of unclassified patients with ILDs both in earlier and later years of the study period argues against major diagnostic improvements over time. Finally, the coding practice may have become more meticulous during the last decade due to more focus on ILD.

Some of the observed changes in subgroups are of interests. The observed predominance of ILD among male patients has previously been described [5,6,9]. It could stem from higher occupational exposure to risk factors [9] or to higher smoking frequency among men. Among ILD subcategories, the highest average increase in incidence was observed in the non-specific category "Respiratory disorders in diseases classified elsewhere". This category includes, however, not only rheumatoid lung disease and ILD associated with diffuse connective tissue disorders but also respiratory disorders in infections and other illnesses. Decreased ILD incidence for most subcategories of pneumoconiosis may reflect reduced use of asbestosis products and safer working environments.

## Conclusion

In conclusion, we found a higher incidence of ILD than reported in previous European studies [5]. The overall IR of ILD showed a clear increase between 1995–2000 and 2001–2005, in particular for ILDs associated with other systemic diseases. These findings underscore the need for improved primary prevention efforts to reduce risk factors associated with ILD.

## Competing interests

Dr Annette Beiderbeck was full-time employee of Glaxo Smith Kline at time of the study. All authors declare they have no conflicts of interest.

## Authors' contributions

JBK coordinated the study, contributed to the analysis of the data and preparation of the paper. SC and PW contributed to the design of the study and the analysis of the data and preparation of the paper. MCG and LP analysed the data and contributed to the design of the study and preparation of the paper. AB and HTS originated the study, contributed to the design of the study and preparation of the paper.

## Pre-publication history

The pre-publication history for this paper can be accessed here:



## Supplementary Material

Additional file 1 **Appendix.** This appendix shows the ICD-10 codes used to identify patients with ILD. Click here for file
