# Supplementary material for: The incidence of interstitial lung disease 1995–2005: a Danish nationwide population-based study
Source: BMC Pulm Med. 2008 Nov 4;8:24. doi: 10.1186/1471-2466-8-24 (PMC2642752; doi:10.1186/1471-2466-8-24)
Supplement: Additional file 1 — Appendix. This appendix shows the ICD-10 codes used to identify patients with ILD. [file 1471-2466-8-24-S1.doc]

**Appendix**

| Code |  |
| --- | --- |
| J60 | Coal workers’ pneumoconiosis |
| J61 | Pneumoconiosis due to asbestos and other mineral fibres |
| J62 | Pneumoconiosis due to dust containing silica |
| J62.0 | Pneumoconiosis due to talc dust |
| J62.8 | Pneumoconiosis due to other dusts containing silica |
| J63 | Pneumoconiosis due to other inorganic dusts |
| J63.0 | Aluminosis (of lung) |
| J63.1 | Bauxite fibrosis (of lung) |
| J63.2 | Berylliosis |
| J63.3 | Graphite fibrosis (of lung) |
| J63.4 | Siderosis |
| J63.5 | Stannosis |
| J63.8 | Pneumoconiosis due to other specified inorganic dusts |
| J64 | Unspecified pneumoconiosis |
| J65 | Pneumoconiosis associated with tuberculosis |
| J67 | Hypersensitivity pneumonitis due to organic dust |
| J67.0 | Farmers´ lung |
| J67.1 | Bagassosis |
| J67.2 | Bird fancier’s lung |
| J67.3 | Suberosis |
| J67.4 | Malt workers’ lung |
| J67.5 | Mushroom-workers’ lung |
| J67.6 | Maple-bark-strippers’ lung |
| J67.7 | Air-conditioner and humidifier lung |
| J67.8 | Hypersensitivity pneumonitis due to other organic dusts |
| J67.9 | Hypersensitivity pneumonitis due to unspecified organic dust |
| J68 | Respiratory conditions due to inhalation of chemicals, gases, fumes and vapours |
| J68.0 | Bronchitis and pneumonitis due to chemicals, gases, fumes and vapours |
| J68.1 | Acute pulmonary oedema due to chemicals, gases, fumes and vapours |
| J68.3 | Other acute and subacute respiratory conditions due to chemicals, gases, fumes and vapours |
| J68.4 | Chronic respiratory conditions due to chemicals, gases, fumes and vapours |
| J68.8 | Other respiratory conditions due to chemicals, gases, fumes and vapours |
| J68.9 | Unspecified respiratory conditions due to chemicals, gases, fumes and vapours |
| J69 | Pneumonitis due to solids and liquids |
| J69.0 | Pneumonitis due to food and vomit |
| J69.1 | Pneumonitis due to oils and essences |
| J69.8 | Pneumonitis due to other solids and liquids |
| J70 | Respiratory conditions due to other external agents |
| J70.0 | Acute pulmonary manifestations due to radiation |
| J70.1 | Chronic and other pulmonary manifestations due to radiation |
| J70.2 | Acute drug-induced interstitial lung disorders |
| J70.3 | Chronic drug-induced interstitial lung disorders |
| J70.4 | Drug-induced interstitial lung disorders, unspecified |
| J82 | Pulmonary eosinophilia, not elsewhere classified |
| J84 | Other interstitial pulmonary diseases |
| J84.0 | Alveolar and parietoalveolar conditions |
| J84.1 | Other interstitial pulmonary diseases with fibrosis (idiopathic pulmonary fibrosis) |
| 84.J 2 | Desquamative interstitial pneumonia |
| J84.3 | Bronchiolitis obliterans organizing pneumonia |
| J84.8 | Other specified interstitial pulmonary diseases |
| J84.9 | Interstitial pulmonary disease, unspecified |
| J99 | Respiratory disorders in diseases classified elsewhere |
| J99.0 | Rheumatoid lung disease |
| J99.1 | Respiratory disorders in other diffuse connective tissue disorders |
| J99.8 | Respiratory disorders in other diseases classified elsewhere |
| D76.0 | Langerhans´ cell histiocytosis |
| D86 | Sarcoidosis |
| D86.0 | Sarcoidosis of lung |
| D86.2 | Sarcoidosis of lung with sarcoidosis of lymph nodes |
